# Understanding discrepancies in perceived importance of patient safety measures between patients and healthcare professionals in perioperative care: An exploratory study

**DOI:** 10.1371/journal.pone.0344802

**Published:** 2026-03-17

**Authors:** Maria de Lacerda Machado, Ana Beatriz Nunes, Dimey Carvalho, Ana Gama, Carola Orrego, Paulo Sousa

**Affiliations:** 1 National School of Public Health, NOVA University Lisbon, Lisbon, Portugal; 2 NOVA National School of Public Health, Comprehensive Health Research Center, CHRC, LA-REAL, NOVA University Lisbon, Lisbon, Portugal; 3 Amélia Leitão Public Health Unit, Western Lisbon Local Health Unit, EPE, Cascais, Portugal; 4 Avedis Donabedian Research Institute, Barcelona, Spain; 5 Universidad Autónoma de Barcelona, Barcelona, Spain; Public Library of Science, UNITED KINGDOM OF GREAT BRITAIN AND NORTHERN IRELAND

## Abstract

**Background:**

Patient safety is a critical concern in perioperative care. This study explores the discrepancies in how patients and healthcare professionals perceive the importance of perioperative patient safety outcome measures, aiming to improve the development of future Core Outcome Sets (COS).

**Methods:**

Qualitative exploratory study using focus groups with healthcare professionals and patients involved in the Core Outcome Set for Patient Safety in Perioperative Care. Data were collected through online mini-focus groups and analysed using thematic qualitative text analysis.

**Results:**

Communication failure emerged as the predominant cross-cutting issue across discussions, particularly in relation to discrepancies in expectations, information exchange, and understanding between healthcare professionals and patients. Three primary reasons for discrepancies in attributed importance of indicators were identified: different targets/focus; knowledge gaps; and varying importance placed on the sense of safety. Patients often emphasized subjective experiences, fears, and emotional impacts, leading them to prioritize quality of life indicators and long-term effects. In contrast, healthcare professionals focused on system-level factors and resource limitations, giving greater weight to technical and physiological outcomes.

**Discussion/conclusion:**

The study findings underscore the need for a more holistic approach in developing COS, balancing technical medical outcomes with patient-centered quality of life measures.

## Introduction

Ensuring patient safety is a critical priority in healthcare, as adverse events (AE) in medical care present a significant global public health concern [1,2]. Estimates suggest that there are 42.4 million injuries per year caused by AE and 22.6 million disability-adjusted life years (DALYs) lost worldwide [3]. In the European Union (EU), between 8% and 12% of patients admitted to hospitals experience AE, with surgical-related effects being among the most common [4–7]. which makes patient safety a public health topic vulnerable to intervention to meet the Sustainable Development Goals (SDGs), especially the third one, ‘Good Health and Well-Being’ [8].

Standardization of practices and outcomes has been recognized as a crucial strategy to improve patient safety. However, the development and implementation of standardized practices often faces challenges, leading to suboptimal outcomes [9,10]. The SAFEST project, funded by the EU Horizon Research and Innovation Programme, aims to address this gap by reducing the incidence of adverse events in perioperative care (before, during, or after surgery) through the implementation of patient-centred, standardized practices across the EU [11].

A key component of the SAFEST strategy is the development of a Core Measure Set (CMS) for Patient Safety in Perioperative Care (CMS-PSPC), comprising structure, process, and outcome measures. This EU-wide CMS was designed to support the assessment of patient safety in routine perioperative healthcare services. By promoting standardization in the measurement and reporting of patient safety, the initiative seeks to improve communication, facilitate benchmarking, and enhance comparability across clinical services and healthcare systems [11–14].

At the core of the CMS is the prioritization of outcome measures, which led to the establishment of a Core Outcome Set (COS) for patient safety in perioperative care. The COS comprises a standardized set of outcomes deemed essential by both healthcare professionals (HPs) and patients for routine measurement and reporting [12]. Importantly, during the development of the CMS-PSPC, discrepancies emerged between the perspectives of patients and HPs regarding the outcome measures. Patient experienced outcomes and quality of life were considered of greater importance by the patients themselves when compared to the responses of healthcare professionals [11,13].

Understanding these divergences in perceived importance of patient safety measures is crucial for ensuring the COS reflects the needs and expectations of all stakeholders. This exploratory study, developed within the SAFEST project explores the differences in perceived importance of perioperative patient safety measures between patients and HPs. By identifying and analysing these discrepancies, this research aims to provide valuable insights to inform the development of effective, patient-centred safety set of outcomes that address the concerns of both key stakeholder groups [15]. Ultimately, this research seeks to bridge the gap between patient and HPs perspectives, fostering a more collaborative approach to improving the monitoring and evaluation of patient safety activities and initiatives in perioperative contexts.

## Methods

This research constitutes an exploratory qualitative study based on thematic qualitative text analysis [16]. Focus groups were conducted to gather rich, in-depth data on participants’ experiences and perceptions. This technique was chosen for its ability to capture complex social dynamics and generate insights through group interaction [17]. The study design, data collection, and analysis procedures were guided by established qualitative research principles to ensure rigor and trustworthiness in our findings, adhering to the Standards for Reporting Qualitative Research (SRQR) and the Consolidated Criteria for Reporting Qualitative Research (COREQ) to enhance transparency and quality in our reporting [2,18].

### Recruitment

The sample was purposively selected from the expert panel involved in the consensus process of the CMS-PSPC, which included a diverse group of stakeholders -namely HPs and patients – previously recruited through other components of the SAFEST project [12,13,16] For this study, recruitment targeted individuals from both groups who had expressed interest and confirmed their availability to participate in online focus group discussions.

Participants were selected based on their involvement in the eDelphi rounds and the consensus process for developing the COS for Patient Safety in Perioperative Care within the SAFEST project [13,19]. Participants included HPs and patients or patient representatives who confirmed their interest and availability, ensuring their ability to engage in the online focus group discussions. A total of 43 HPs and six patients or patient representatives were contacted [14].

As exclusion criteria, individuals who were not involved in the eDelphi process or consensus development were not considered. Additionally, participants who expressed interest but were unavailable for the scheduled focus group sessions or did not respond to follow-up emails were not included.

Email was used to establish communication. Eligible participants’ interest and availability were identified in the first contact, and the focus group schedule and logistics were finalized in the second follow-up contact.

### Data collection

Data collection was conducted through online mini-focus groups using the Microsoft Teams platform, facilitated in English, during July 2024. The focus groups consisted of three to four individuals each and lasted approximately 90 minutes [17].

The concept of mini-focus groups was introduced as a methodological approach, particularly useful when participants possess specialized knowledge and experience on the subject matter [16]. Mini-focus groups are employed when there are constraints on participant availability for scheduled focus groups [16]. Furthermore, online groups are recommended to consist of four or five participants per session to facilitate effective discussion and interaction [20]. This methodology was utilized to ensure that expert insights were gathered efficiently while accommodating participants’ scheduling constraints, thereby optimizing the data collection process.

The sense of belonging to a group enhances cohesiveness and creates a safe environment for sharing information [16]. To facilitate effective discussions, group members should feel comfortable and engaged [17]. Therefore, carefully selecting group members and using homogeneous groups, where participants share similar characteristics such as gender, age range, ethnic and social class background, is crucial to ensure meaningful interactions and a more dynamic discussion [17]. Additionally, it is advisable that participants do not know each other to encourage honest and spontaneous responses [17]. In order to prevent the potential power dynamics that could occur in joint focus group settings and to guarantee open and honest discussions, separate focus groups were held for HPs and patients.

During the development of the COS for Patient Safety in Perioperative Care within the SAFEST project, experts were asked to rank each measure based on its perceived importance and measurement feasibility [12,13]. In this study, only measures rated as “Critical for inclusion” by at least 50% of patients were initially selected. Subsequently, the level of agreement between patients and healthcare professionals (HPs) was examined, and measures were retained when more than 50% of the “Critical for inclusion” ratings originated from patients. The selected measures were then grouped into four overarching themes to facilitate focused and meaningful discussions: (1) Communication and Information Exchange in Patient Diagnosis; (2) Long-Term Outcomes, Recovery, and Functional Status; (3) Psychological and Emotional Recovery; and (4) Pain Management and Well-being of Patients and Caregivers (appendix 1).

For the conduction of the focus group discussions, a semi-structured guide (appendix 2) was developed and tested in two pilot focus groups, which included both healthcare professionals and patients. The guide was adapted according to the feedback from these pilot groups. The data from the pilot focus groups were not included in the final analysis. Additionally, a brief questionnaire was applied to collect participants’ sociodemographic information (appendix 3).

Focus groups were conducted by a moderator from the research team (MdLM), and another research team member was responsible for taking notes and logistics (ABN) [16]. These responsibilities were carried out by the same people in every session.

### Data analysis

Focus group discussions were recorded and automatically transcribed, with manual adjustments applied to the transcripts [21]. The transcriptions were not returned to participants for review or corrections. Thematic Qualitative Text Analysis was employed to define the themes within data [22]. In the first phase, the text was analyzed, and key passages were highlighted to code the main deductive thematic categories defined [22]: Perspectives on discrepant opinions between HPs and Patients in general; Perceptions on discrepant opinions between HPs and Patients regarding indicators related to Communication and Information Exchange; Perceptions on discrepant opinions between HPs and Patients regarding indicators related to Long-Term Outcomes and Recovery; Perceptions on discrepant opinions between HPs and Patients regarding indicators related to Psychological and Emotional Recovery; Perceptions on discrepant opinions between HPs and Patients regarding indicators related to Pain Management and Well-being of patients and caregivers; Perspectives on the importance of studying disparities and Perceptions on the variation of discrepancy of opinions between HPs and patients across professional groups. In the second phase, inductive subcategories were identified, emerging during the initial coding process. These subcategories explained the reasons behind different perceptions of the indicators, primarily based in experiences and needs [22]: Different target/focus, Subjectivity of experience, Personalization, Knowledge and information gaps, Sense of safety, System protocols and resources, Human resources, Fears and uncertainty, Emotional impact, Expectations, Factors beyond HPs control, Pain/subjectivity of pain and Drugs consequences.

MAXQDA 2022 software was used for both phases of coding [22]. The transcripts were coded and reviewed by one researcher (MdLM), and two researchers (ABN, DC) independently reviewed the coding. A category–based analysis was subsequently conducted, considering the frequency of comments, that is the number of times a reason was mentioned [17,22]. This analysis evaluated the primary reasons for each main category, the correlation between categories and subcategories, and compared the reasons cited by HPs and patients.

An interaction analysis and micro-interlocutor analysis were also conducted to explore commonalities on the themes [16]. This involved an individual analysis of each participant and their contribution to each theme, allowing for determining the proportion or level of similarity within the themes discussed in the focus groups. Contributions were analysed across three participant groups: two groups of healthcare professionals (G1 and G2) and one group of patients (G3). The coding system used classified participant responses as either 1 (indicating agreement with and contribution to the theme) or 0 (indicating agreement without further contribution). This approach allowed us to examine both the participants’ agreement and the depth of their engagement with the thematic categories. The counts provide valuable insights not only into the degree of consensus or dissent but also into response patterns among the participants, thus avoiding overgeneralization [16]^.^

### Reflexivity

The focus groups were moderated by the principal author (a dentist) and co-moderated by a co-author (a public health physician). Our healthcare backgrounds influenced our approach to the study, as we constantly navigated the tension between our professional perspectives and participants’ lived experiences, especially challenging when moderating the patient group. As young female researchers interacting with predominantly older participants, we experienced shifting power dynamics. While age differences could have created distance, they often allowed participants to adopt an ‘educating’ role, particularly when discussing long-term healthcare experiences. The coding process presented challenges in distinguishing between personal grievances and systemic issues causing disparities. Our healthcare backgrounds sometimes made it difficult to separate valid system criticisms from individual complaints, necessitating frequent team discussions to check our biases. However, we observed that participants were generally open and forthcoming with their views, including critical perspectives on HPs and systems. Throughout the analysis, we remained aware that our interpretation of the data is inevitably a reinterpretation of the participants’ positions. Our construction of meaning may extend beyond the participants’ intentions, and they might not fully agree with our analysis. We approached this study from our specific perspectives as HPs committed to improving patient care and patient participation in research, within the context of the SAFEST project’s research agenda.

### Ethical considerations

The SAFEST project has received approval from the IDIAP Jordi Gol’s Research and Ethics Committee [11]. Written informed consent for participation in the focus groups and for audio and video recording was obtained from all individual participants included in the study (appendix 4). Participants were also informed that their involvement was voluntary and that they could withdraw from the study at any point [11].

The storage and management of data adhered to EU Regulation 2016/679 [23]. Only authorized members of the research team have access to the safely stored participant data. The information gathered will be permanently erased after the project is finished, which is expected to happen in June 2026.

## Results

### Characteristics of the participants

The study sample comprised 11 participants across three focus groups (Table 1). Two focus groups consisted of eight HPs (four participants in each group), while a separate focus group included three patients or patient representatives. The participants were all from different countries, including Norway, Italy, the Netherlands, Spain, Greece, Lithuania, the UK, Croatia, Estonia, Spain, USA, and Italy. The sample was predominantly female (n = 6) with a balanced age distribution. It’s worth mentioning that 15 additional individuals expressed interest in the study but did not complete the recruitment process, potentially indicating barriers to participation that may warrant further investigation.

**Table 1:** Demographic Data.

Participant	Age Group	Gender	Level of Education	Occupation	Profession	Area of expertise	Submitted to surgery ≤ 5 years ago
**Focus Group 1**							
1	55-74	F	Postgrad	Healthcare Professional	Medical Doctor	Surgery	–
2	55-74	M	Postgrad	Healthcare Professional	Medical Doctor	Other	–
3	55-74	F	Postgrad	Healthcare Professional	Medical Doctor	Anesthesiology	–
4	55-74	M	Postgrad	Healthcare Professional	Nurse	Other	–
**Focus Group 2**							
1	55-74	M	Postgrad	Healthcare Professional	Medical Doctor	Surgery	–
2	35-54	F	Postgrad	Other	–		–
3	55-74	F	Postgrad	Healthcare Professional	Medical Doctor	Anesthesiology	–
4	35-54	F	Postgrad	Healthcare Professional	Medical Doctor	Anesthesiology	–
**Focus Group 3**							
1	35-54	M	Prefer not to answer	Patient/Patient Representative	–	–	Yes
2	55-74	F	Postgrad	Patient/Patient Representative	–	–	No, but a direct family member was
3	35-54	M	Postgrad	Patient/Patient Representative	–	–	Yes

### Perspectives on discrepancies in perceived importance of patient safety indicators between patients and HPs

Table 2 shows the main deductive thematic categories identified through Thematic Qualitative Text Analysis.

**Table 2:** Codes Assigned: Main Thematic Category.

Codes Assigned- Main Thematic Category	
C1-Perspectives on discrepant opinions between HPs and Patients in general	This category explores the overall differences in viewpoints between healthcare professionals and patients regarding perioperative care priorities
C2-Perceptions on discrepant opinions regarding Communication and Information Exchange	This category focuses on how healthcare professionals and patients differ in their views on the importance and effectiveness of communication and information sharing during the perioperative process.
C3-Perceptions on discrepant opinions regarding Long-Term Outcomes and Recovery	This category examines the differences in how healthcare professionals and patients perceive and prioritize long-term outcomes and the recovery process
C4-Perceptions on discrepant opinions regarding Psychological and Emotional Recovery	This category addresses the disparities in how healthcare professionals and patients view the importance of psychological and emotional aspects of recovery
C5-Perceptions on discrepant opinions regarding Pain Management and Well-being	This category explores the differences in how healthcare professionals and patients perceive the importance and effectiveness of pain management strategies and overall well-being during the perioperative period
C6-Perspectives on the importance of studying disparities	This category focuses on how both healthcare professionals and patients view the significance of researching and understanding the differences in their perspectives on perioperative care
C7-Perceptions on the variation of discrepancy across professional groups	This category examines how the differences in opinions between healthcare professionals and patients may vary across different healthcare specialties or professional role

Table 3 presents the inductive codes that emerged during the analysis. These codes explain the reasons behind the differing perceptions, based on participants’ experiences and needs.

**Table 3:** Codes Which Emerged from the Focus Groups: Reasons for different perspectives.

Codes Which Emerged from the Focus Groups- Themes: Reasons for different perspectives	
T1-Different target/Focus	Healthcare professionals tend to focus on technical and medical aspects, while patients prioritize overall well-being and quality of life.
T2-Subjectivity of experience	Patients’ perceptions are heavily influenced by personal experiences and emotions, while professionals view cases more objectively
T3-Personalization	Patients desire tailored communication and information specific to their individual cases
T4-Knowledge and information gaps	Significant disparity in medical knowledge between healthcare providers and patients, influencing care priorities and healthcare professionals may provide excessive technical details, while patients feel they lack practical recovery information.
T5-Sense of safety	Refers to the patients’ perception of security and trust in the healthcare system during the perioperative process, which significantly influences their prioritization of outcome indicators, as they seek reassurance about their well-being, the effectiveness of communication, and the competence of the surgical team.
T6-System protocols and resources	Healthcare professionals are constrained by system protocols and resource limitations, which may not align with patients’ expectations
T7-Human resources	This subcategory refers to the availability, expertise, and allocation of healthcare professionals involved in perioperative care. It includes factors such as staffing levels, skill mix, and workload that can impact the quality of care and patient outcomes.
T8-Fears and uncertainty	Patients grapple with fears about the future that may not be fully addressed by healthcare providers.
T9-Emotional Impact	Patients experience significant emotional stress that may be underestimated by healthcare professionals
T10-Expectation	Mismatch between patients’ recovery expectations and reality, not always fully communicated by healthcare professionals
T11-Out of HPs control	Factors related to the disease progression, prognosis, and patient circumstances that healthcare professionals cannot directly influence or change. This includes aspects such as the natural course of the illness, genetic predispositions, or socio-economic factors that impact long-term outcomes and recovery.
T12-Pain/Subjectivity of pain	Patients’ experiences of pain and discomfort may not align with healthcare professionals’ clinical assessments. Additionally, pain perception varies significantly among patients; what one patient considers severe pain might be perceived as mild discomfort by another.
T13-Drug Consequences	This subcategory encompasses the effects, both intended and unintended, of medications used in perioperative care. It includes considerations of drug efficacy, side effects, interactions, and long-term impacts that may influence patient experiences and outcomes

The results are presented by category, highlighting the reasons underlying the discrepancies in measure evaluations between healthcare professionals and patients. The viewpoints presented are contextualized and supported by illustrative quotations as presented in Table 4.

**Table 4:** Results synthesis.

C1 - Perspectives on Discrepant Opinions between HPs and Patients in general		
Themes	Reason Explain	Quotes
Different Focus: Technical Outcomes vs. Quality of Life Measures (T1)	HPs often prioritize technical and medical outcome indicators, concentrating on physiological parameters and immediate surgical success.	G2P4: “We focus, and we are stressed about physiological parameters.”
	Patients tend to prioritize quality of life indicators that reflect their overall well-being post-surgery	G3P2: “Patients are interested in, you know, their global well-being, how they want to be. Well, as a whole, so they don’t just want the operation to be a success.”
Knowledge and information gaps and Personalization (T3 and T4)	HPs often assume a certain level of understanding from patients regarding their conditions and procedures, which may not reflect reality. This can lead to a lack of personalized information tailored to individual patient needs.	G1P1: “There is also a very big power imbalance about this knowledge between the doctors and the patients.”
System protocols and resources (T6)	HPs are bound by organizational protocols that can constrain their focus on patient-centered outcomes.	G2P2: “There are also organization protocols that have to respect and goals to achieve.”
	This adherence to protocols can create stress among healthcare professionals	G2P4: “Healthcare nowadays are under immense stress... we expected, as healthcare professionals, to deliver better care with the same amount of money invested.”
Patient Concerns: Fears, Uncertainty, and Sense of Safety (T8 and T5)	Patients frequently express fear and uncertainty regarding surgical procedures and their outcomes. The feeling of safety is crucial for patients in perioperative care, as it encompasses their need for reassurance and trust in the healthcare system, influencing their prioritization of outcome indicators related to quality of life and emotional well-being, rather than just the technical success of surgical procedures.	G3P1: “We live with the uncertainty about the future and we live with panic because it’s dramatic for us.”
**C2 - Perceptions on Discrepant Opinions Regarding Communication and Information Exchange**		
**Themes**	**Reason Explain**	**Quotes**
Knowledge and information gaps and Patient Concerns (T1,T3, T4, T8 and T10)	A key factor in these discrepancies is the imbalance of medical knowledge between HPs and patients. HPs often underestimate patients’ concerns about certain aspects of surgery due to this knowledge gap.	G1P3: “Whereas for the patients of course they know that the heart is very important for the body, so they might be more concerned out of what they know and from what we know.” G1P1: “They think that the heart is the problem in many ways but, well, it’s the perioperative care. And that’s why we have to, well, give them also more information, especially when you build the care pathways.”
	HPs noted that patients sometimes request additional tests or information due to a lack of trust in the system.	G2P2: “Patients don’t trust the system. So they tend to request more information uhmm out of uhmm lack of secure, lack of safety or mistrust.”
	From the patient perspective, this desire for more information is linked to shared decision-making and risk minimization.	G3P1: “I think it’s important the shared decision making. I mean in you need to have all the information about the operation, the risk obviously, but the benefits also and in order to take the better decision for you is expectative because, repeat myself, it’s my mantra and it’s my life, it’s my quality of life.” G3P3: “The more experience you have, the more detailed questions you have and the more, actually, the doctors need needs to have a time to explain everything...”
Patient Sense of Safety (T5)	HPs believe that team satisfaction and cohesion contribute to patient safety and confidence.	G1P2: “I think if they see a surgical team that is uhmm is is close to team working, are satisfied, they would, they would be more trustworthy in that team. And probably they will approach surgery a little bit better without, you know, any fear...”
	Patients seem to focus more on team agreement and a non-intimidating culture in the operating room	G3P2: “One is that you want everyone to be in agreement. Some of the worst surgical errors I’ve seen have been where the team was not in agreement and you know somebody was, you know, persuaded to go along against their better judgment.”
**C3 - Perceptions on discrepant opinions regarding Long-Term Outcomes and Recovery**		
**Themes**	**Reason Explain**	**Quotes**
Different Focus: Technical Outcomes vs. Quality of Life Measures (T1,T3,T8)	HPs tend to focus on immediate physiological aspects and medical success of the surgery, while patients are more concerned with long-term impacts and recovery.	G1P2: “If the patient is still alive, first of all. ((laughs))” and “had to manage pain and other considerations. “ G1P4: “But the surgery, for for the surgery team, was okay, we we- they removed the cancer, there are no complications sinc- at the, at the operating room. It’s the other view of the, of the question.”
	HPs often overlook the entire patient process due to time constraints and focus on immediate care.	G1P4: “We don’t think about th- all the process of the patient, because we have another surgery in 10 minutes and we must to prepare and we must to receive the the next patient.”
	Patients prioritize indicators related to their quality of life and socioeconomic circumstances, which HPs might overlook	G2P2: “For patients these are very important because they hide a lot of concern, concern about dependence to other people, concern about children, return to work, lost of productive time and a lot of issues. Humm and, of course, all these are also depending on the financial and the socioeconomic circumstances of the patient.”
Knowledge and information gaps and Human Resources (T4,T7)	Patients express a need for more information about recovery and long-term impacts, as well as ongoing support.	G3P2: “I think not knowing what recovery is expected to be like is really critical for patients and they usually don’t know because nobody wants to talk about that.” and “To have follow-up and nurses who follow-up or someone from the surgical office, you know, who follows up to make sure everything is alright... it’s very helpful. People need to have someone, to call and to rely on, have a number and then have someone who calls.”
Out of HPs control (T11)	HPs may not consider certain indicators due to uncertainty about long-term outcomes or factors outside their control.	G2P2: “All these aspects sometimes are not easy to answer because the health professionals don’t know them, don’t know how each patient will react and what uhmm the next- the future of each patient will be.” G2P3: “While we see, we cannot uhmm uhmm we cannot modificate this because they, these are part of the disease.”
**C4 - Perceptions on discrepant opinions regarding Psychological and Emotional Recovery**		
**Themes**	**Reason Explain**	**Quotes**
Different Focus: Technical Outcomes vs. Quality of Life Measures (T1)	Patients often equate immediate post-surgery awareness with successful outcomes.	G1P1: “He awakens after surgery and he manages to realize that he’s seeing other people, that he’s seeing other uhmm another environment, that he awakened, maybe he would might say, might, might say on his perspective, on he or she’s perspective, that the surgery went okay, that everything went okay, that there were no complications, that there were nothing, I’m alive, you know, I’m alive, look at me, I woke it up so I’m alive. Uhmm And I think that is important for the patient.”
	HPs tend to prioritize surgical success over psychological and cognitive factors.	G2P1: “I would as well agree very much with with with with that definition that they seem to us so much secondary, those those outcomes, really. I mean, it doesn’t mean that they are not important. But but I but but but, as professionals, we would very much treat them as secondary outcomes, very much secondary.” G2P2: “Then the primary goal is to save the patients and have a a successful uh surgery.”
Limited Human Resources and Out of HPs control (T7, T11)	HPs acknowledge the lack of mental health resources in some healthcare systems.	G2P2: “In some countries access to mental health professionals in the public health se- in the healthcare system is not easy, they’re not available. So, there could be also some concern about whether there will be adequate, trained and appropriate professionals to support in these cases, or if they would be managed by other professionals or less adequately handled if they develop such symptoms.”
	HPs face difficulties in measuring mental and cognitive states before and after surgery.	G2P4: “But the neuropsychological performance, mental health and psychological recovery, we don’t measure it pre-op, uhm we don’t have the means to measure it post-op. I don’t think that uhmm, I will speak as an anaesthetist, but I think partially is in the surgeons, uhm they cannot modulate that, these these aspects.”
Drugs Consequences and Emotional Impacts (T13 and T9)	Patients prioritize psychological factors due to concerns about medication effects and the emotional impact of physical changes.	G3P2:“The drugs they are on that can cause terrible hallucinations.” and “It’s critically important and and obviously surgeons are not involved in this at all, and I think they need to be, you know something people need to be to learn about and be trained about.” G3P3 expressed: “You lost your hand, you know, you just it’s it’s the probably it’s your your mental health that needs to be supported and that’s why you need to maybe talk to someone who is already went through this or you have to have the psychologist or you need more help. And and this is really important because if they send you home, you’re fine, but your mind is not. Then you will not go out, you probably will not- Your relationships will maybe even end because if you are not accepting your new body, you can’t show it to others. And then it’s disaster.”
**C5 - Perceptions on discrepant opinions regarding Pain Management and Well-being**		
**Themes**	**Reason Explain**	**Quotes**
Different Focus: Technical Outcomes vs. Quality of Life Measures (T1)	Healthcare professionals possess a broad view of medical procedures, observing various interventions throughout the day. In contrast, patients evaluate their experiences from the perspective of their specific illness.	G1P1: “Yes, I I also think that the difference could be, well, we are looking in at, well, with a, with a, well, a wide view and just we’ve seen a lot of things and we have all kind of different uhm yeah perspectives on the different procedures we see during the day and during the weeks. The patient is the one with the disease and the patient who answers is looking from the perspective of him, his or her disease. [I think] that’s the difference.”
Patient Concerns: Pain and Emotional Impact (T9 and T12)	Patients’ primary concerns after surgery revolve around pain management, nausea, vomiting, and returning to normal life.	G1P2: “I think these two or major three aspects, the patient evaluates very very seriously because it’s what he or she doesn’t want to experience after surgery. So they’re mainly interested in not having pain afterwards. In fact, sometimes they asked me- Well the question, most often, that occurs to- from my patients to me, for example is, Will I have severe pain after and will I be able to eat again? Will I be able to do my normal activities?”
	Many patients face emotional challenges due to a lack of available caregivers, which heightens their concern about pain management.	G1P1: “And also about the network. When you see a lot of caregivers, this is a burden for patients when they don’t have caregivers or they know that their children live away 200 kilometers, do their jobs and everything.”
	HPs may not fully recognize the level of concern patients have regarding pain management due to their knowledge and control over procedures.	G2P2: “On the other side, professionals already know. So everything, all these aspects are under control. They know what’s after, they know what follows. So this is why it’s so, not so highly important as it is for patients.”
	Patients often experience fear and anxiety due to limited access to effective pain relief therapies.	G2P2: “But generally we don’t see much of palliative care services available in Europe. So we see that the pain is not easily managed, especially in certain situations and certain diseases. And this has generated fear, concern.”
Personalization and Knowledge and Information Gaps (T3 and T4)	Patients emphasize the need for individualized treatment plans and clear communication regarding pain management strategies	G3P3: “I think it’s all also coming down to the counseling in a language I understand because if the doctor, the nurse, whoever talk to you before the operation... they need to also like ask questions and understand if I understood what he or she was telling me.”
Drugs Consequences (T13)	Patients worry about the effects of medication on their recovery process.	G3P2: “All of these things involve drugs. Either they’re caused by drugs or they need drugs to fix them... in my experience... healthcare providers in general... don’t know enough about the drugs that they’re using.”

### Comparison of perspectives and thematic categories

**Fig 1:**
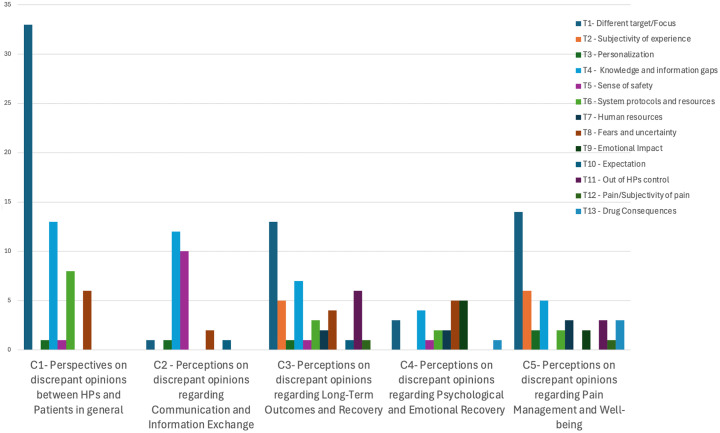
Comparison of Perspectives and Thematic Categories.

The analysis of focus group discussions revealed five main categories (C1 to C5) representing thematic groupings. As illustrated in (Fig 1), category C1 showed the highest number of responses, particularly associated with theme T1 (Different target/Focus), indicating its widespread discussion. C2 also had a high number of responses, with themes T4 and T5 being most frequent. C3 displayed a more balanced distribution of themes, with T1 and T4 being prominent. In C4 with similar participation from T8 and T9, followed by T4. C5 was similar to C3, with a higher concentration in T1, followed by T2 and other themes. Notably, T1 was consistently the most addressed theme across all categories, suggesting it was a central topic in the collected responses. Themes T2 and T4 also appeared in various categories, albeit less frequently, indicating their lesser but still relevant importance. The high frequency of T1 across categories may reflect an important concern or consensus among participants. Conversely, the lower frequency of themes like T6 and T7 suggests they were less relevant to most participants or mentioned by fewer individuals. The bar graph illustrates a heterogeneous distribution of responses within each category, with some themes being discussed more extensively (e.g., T1) and others more sporadically. The predominance of theme T1 and T4 across all five categories suggests it may be a key point for more detailed analysis, while the other themes help contextualize the diversity of responses.

**Fig 2:**
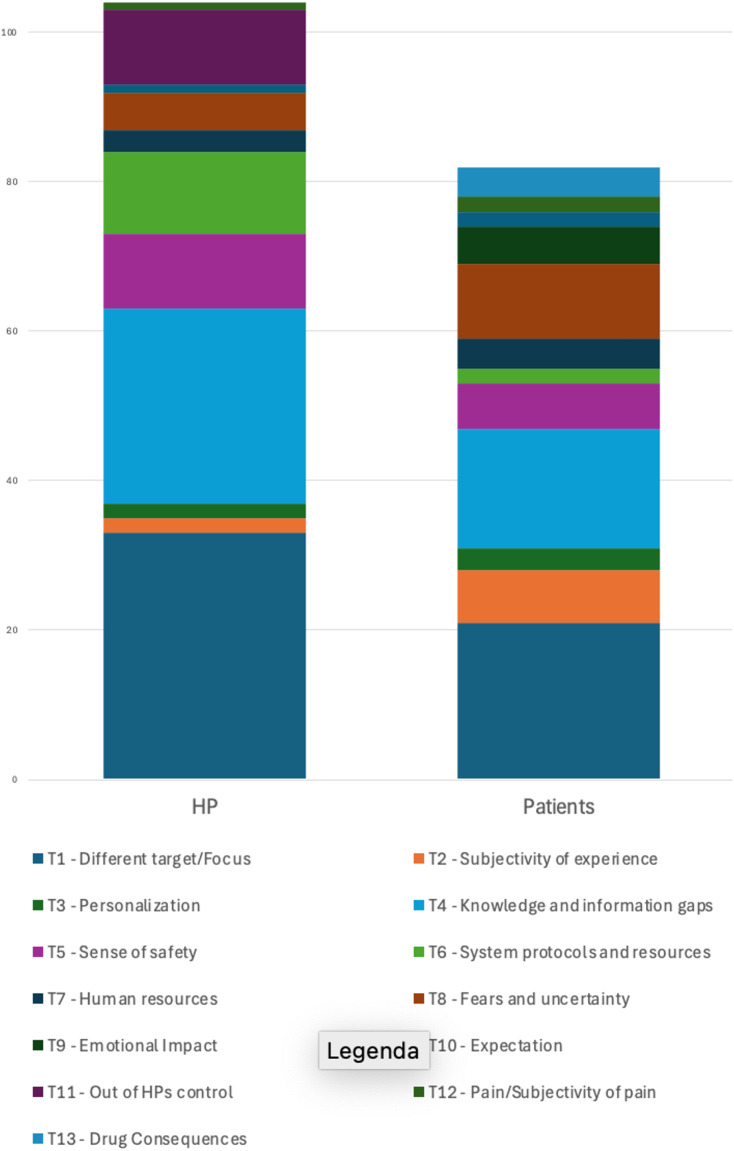
HP vs. Patients.

The analysis revealed distinct patterns in the distribution of themes (T1 to T13) between patients and HPs, as shown in (Fig 2|). Both groups prominently featured T1, T4 and T5 as the central themes, indicating its universal importance in perioperative care. For patients, T1 was followed in importance by T2, T8, T9 and T13, with a relatively balanced distribution among other themes. This suggests that patients have focused concerns on a few main themes (T1, T4, T5) while also addressing various other topics. HPs, while also prioritizing T1, T4, T5, showed a different pattern in their secondary themes. They placed greater emphasis on T6 and T11, with T8 being less prominent compared to patients. This pattern indicates a more diverse and specialized perspective among professionals, possibly focusing on practical or organizational approaches. While both groups considered T1 most important, patients seemed more concerned with themes related to their care experience (T2, T8, T9, T13), whereas HPs emphasized themes potentially linked to care practice and implementation (T6, T11). This analysis reveals shared central concerns (T1) but notable differences in secondary interests, with patients concentrating on practical and emotional issues, while HPs tend to value more technical or operational aspects.

### Importance of studying disparities

The study of disparities between HPs and patients in scoring measures, and the importance of including patients in COS development processes, is crucial for several reasons. Patients offer unique perspectives that can redefine value in healthcare, as G1P1 noted: “We have to not really redefine value like Porter [the author Michael Porter] did but redefine the value for the patient.”. Their involvement ensures that outcomes reflect what matters most to patients, beyond just clinical success. As G2P4 stated, “We need to know what kind the patients want us to deliver. So uhh and it’s, it goes beyond waking up at the end, like being- having a successful anaesthetic by waking up at the end, or resection of tumor if we’re talking about cancer.” Patients can highlight aspects of care that HPs might overlook, such as long-term quality of life impacts and emotional preparation for treatment. Including diverse patient voices, especially those with lower health literacy, is essential for addressing health inequities: “Patients with low healthcare skills, their life and their quality of life changes when they’re 57 years old. Well, they’re well educated, then the problems only start when they’re 72 years old. That’s 15 years of change.”(G1P1). Patient involvement also improves communication and expectation management, as G2P4 noted: “Only by listening to patients in this kind of setting we will improve our communication with our day-to-day patients and manage the expectations and and prepare them properly.” Ultimately, patient inclusion in COS development aligns with the principles of patient-centered care and value-based healthcare: “So if we don’t involve the patient in all of this, we’re probably not gonna get value-based healthcare.”(G1P2).

### Variation across professional groups

Anesthesiologists tend to score perioperative outcome measures more similarly to patients compared to other HPs, particularly surgeons. This alignment stems from their focus on patient experience, research priorities that directly impact patient quality of life, and recognition that their techniques can influence patient-prioritized outcomes. As one anesthesiologist noted, “Some of these indicators are actually primary outcomes of anaesthesia research for the last 30 years.” (G2P4). They view the operating room as part of a larger care pathway, aligning with patients’ holistic view of treatment: “When you look at care pathways, the operation is a, well, well, the operating room is a shared resource and it’s an aspect of the treatment.”(G1P1). Anesthesiologists emphasize patient awareness and involvement in care, stating, “I would prefer that the patient not only asks, Who is gonna operate me, but I would also prefer for patient would ask me, Doctor, Who will be the anaesthesiologist that is gonna put me into sleep during the operation.”(G1P2). While surgeons may focus on technical outcomes, anesthesiologists consider a wider range of patient-centered outcomes: “These are less related to purely surgical outcomes, which is probably this disease resection if we talk about cancer, uh wound healing and physiological parameters post operatively, and exit from the hospital, and so overall survival, because these are their their indicators for their studies.”(G2P4). This alignment highlights the importance of considering multiple perspectives in developing comprehensive patient safety set of measures.

### Interaction and micro-interlocutor analysis

**Fig 3:**
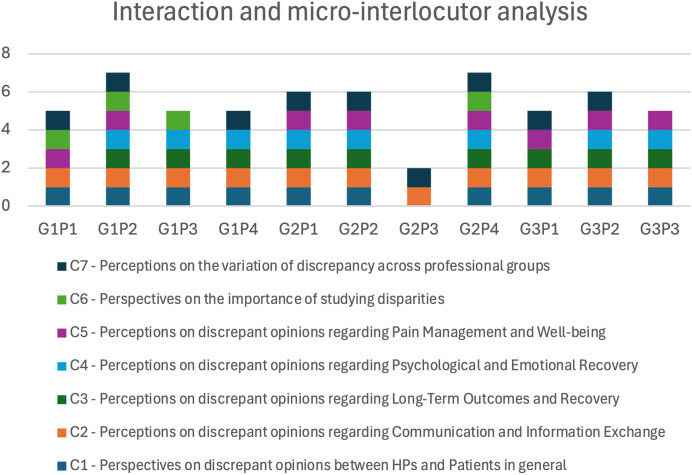
Interaction and micro-interlocutor analysis; G- refers to the focus group number; P- refers to the participant number; E.g- G1P1: Group 1 first participant.

The focus group discussions were categorized into seven thematic categories, each represented by a different color in the bar chart shown in (Fig 3) (C1 to C7).

In the HPs’ groups (G1 and G2), participants showed varied levels of engagement across the colour-coded thematic categories. In G1, all participants (G1P1, G1P2, G1P3, G1P4) actively engaged with multiple categories. G1P2 demonstrated a particularly significant involvement, not only agreeing with the themes but also providing substantive contributions.

In G2, participants also showed high engagement across several categories indicating a broad interaction with the themes. G2P3, however, contributed to fewer categories but still agreed with the primary themes discussed by the group.

The patient group (G3) showed more balanced contributions across the thematic categories. G3P2 contributed to a wide range of themes. G3P1 and G3P3 participated more moderately, showing agreement with several themes but contributing less actively compared to the other participant. These participants provided valuable input on these themes, reflecting their personal experiences.

This comparison highlights the differences in how HPs and patients engage with key themes and within the focus group. Although there were participants who spoke less, overall, everyone actively contributed, which may indicate that they felt safe and comfortable expressing their opinions.

## Discussion

The predominant cross-cutting issue across all discussion categories was communication failure, a finding that underscores the critical importance of effective dialogue in perioperative care. Both patients and HPs identified three primary reasons for discrepancies, observed in the priorization of measures during the development of the COS: different targets/focus, gaps in information and knowledge, and the varying importance placed on the sense of safety.

HPs tend to prioritize technical and physiological outcomes, focusing on immediate surgical success and measurable clinical measures. In contrast, patients emphasize quality of life measures and the long-term impacts on their overall well-being, including social, emotional, and functional aspects of recovery. This difference in perspective highlights an information gap between these groups that significantly influences how each interprets and prioritizes outcomes.

Patients frequently cite reasons tied to the subjectivity of their experience, such as fear and uncertainty surrounding the surgical process, the emotional impact of undergoing a medical procedure, and concerns about medication side effects and long-term consequences. These patient-centric concerns often revolve around their ability to return to normal daily activities and maintain their quality of life post-surgery. On the other hand, HPs more frequently highlight system-level factors such as adherence to established protocols, resource constraints within the healthcare system, and events beyond their immediate control. This divergence in focus naturally leads to differing outcome prioritization between the two groups.

Interestingly, among HPs, anesthesiologists’ ratings most closely aligned with those of patients. This alignment can be attributed to the fact that many key indicators measures prioritized by patients, such as pain management, nausea control, and immediate post-operative recovery, are directly influenced by anesthesia practices. Consequently, anesthesiologists often serve as key evaluators of these patient-centric outcomes, bridging the gap between clinician and patient perspectives [24,25].

The review on patient satisfaction with perioperative anaesthetic care found that factors such as postoperative pain, procedure duration, and patient-physician relationships significantly impact patient satisfaction [25]. Since anesthesiologists are more aligned with these measures, which are closely linked to their clinical practices, they may be key stakeholders in measuring them, especially as they become integrated into a Core Outcome Set (COS). This underscores the potential for anesthesiologists to play a role in bridging the gap between clinician and patient perspectives as referred in Tylee et al. 2020 [24].

Our study’s key finding, which was consistent across all discussion categories, aligns closely with Gobbo et al.’s (2020) research on patients’ experiences and needs during perioperative care [26]. Both studies emphasize patients’ strong desire for detailed, personalized information, particularly regarding recovery processes and long-term impacts. Gobbo et al. (2020) noted that patients felt HPs often assumed they knew things they were not actually informed about, which corresponds to our observation of HPs underestimating the importance of measures that reflect patients’ concerns about specific aspects of surgery [26]. The need for ongoing support throughout the care journey, identified in our study, is echoed in Gobbo et al.’s finding that patients desired a “HPs of reference” across the entire perioperative process [26].

Patients, particularly those with prior experience, express a strong desire for detailed information and explanations, especially regarding recovery processes and long-term impacts [26,27]. Patients want to be involved in decision-making, and for that, they need to be better informed. The lack of trust in the healthcare system sometimes prompts patients to request additional tests or information, which HPs may not anticipate. Effective communication is essential for shared decision-making and patient-centered care [27]. In fact, one study revealed that 23% of patients did not receive an explanation of their health problem when discharged from the emergency department [28].

HPs felt that insufficient patient information often leads to less appropriate care being selected. This discrepancy in information needs and provision contributes to the different prioritization of outcome measures between HPs and patients, emphasizing the importance of bridging this communication gap to improve patient care and satisfaction.

For patients, the feeling of safety is essential in perioperative care, reflecting their need for reassurance and trust in the healthcare system. This need influences their prioritization of outcomes related to quality of life and emotional well-being, beyond the mere technical success of surgery. HPs acknowledge that team satisfaction and cohesion are critical in enhancing patient safety and confidence as also noted in Dy’s (2016) article [27].

The main differences in reasoning stem from the fact that patients frequently refer to subjective experiences, fear and uncertainty, emotional impact, and the consequences of medications. In contrast, HPs often cite system protocols and resource limitations as reasons for their outcome prioritization. Additionally, certain factors that are important to patients are beyond the control of professionals, which leads them to deem these aspects less relevant to be systematically assessed in perioperative patient safety interventions.

For HPs, strict adherence to organizational protocols can sometimes limit their focus on patient-centered outcomes, causing stress, particularly when patients request additional tests or information due to a lack of trust in the system [29]. From the professional perspective, our results suggest that decision-making is a multifactorial process, as referred in Ebben et al (2017) is influenced by the professional, the patient and their relatives, the healthcare system, and available support tools [30].

Fear, uncertainty, emotional impact, and the consequences of medications were prominent concerns for patients in our study. Gobbo et al. (2020) noted that patients often express more fear about anaesthesia than the surgical procedure itself, highlighting the importance of having nursing staff and family members present during care [24,26,31]. These findings underscore the necessity of addressing not only the technical aspects of care but also the emotional and psychological dimensions of the perioperative experience when evaluating perioperative patient safety.

### Importance of including patient perspectives

Our findings underscore the critical importance of integrating patient perspectives into healthcare value assessment and COS development. Patients offer unique insights into living with the effects and conditions of treatments that can redefine value in healthcare, ensuring that outcomes reflect what matters most to them beyond clinical success [32,33]. Their involvement highlights aspects of care that HPs might undervalue, such as long-term quality of life impacts and emotional preparation for treatment [29,33]. Including diverse patient voices in the development of COS, especially those with lower health literacy, is essential for addressing health inequities and improving communication and expectation management [32]^.^ Patient participation in COS development increased the likelihood of inclusion of outcomes measuring the subsequent impact on the person’s life (including physical, cognitive, emotional, social and role functioning).

Barrington (2021) findings align with our results, noting that including patients in Delphi processes for COS development allows for a more comprehensive view, with patients often ranking themes differently than HPs [34]. Gorst (2016) further emphasizes the relevance of public involvement in COS development for comparative effectiveness research, where long-term patient-centered outcomes are critical endpoints [35]. Patient participation in COS development increased the likelihood of inclusion of outcomes measuring the subsequent impact on the person’s life (including physical, cognitive, emotional, social and role functioning), findings from Kearney’s study [36].

Additionally, the growing implementation of Patient-Reported Outcome Measures (PROMs) in healthcare systems demonstrates the increasing recognition of the patient’s perspective in assessing healthcare value and system performance [37].

### Implications for practice and COS development

The discrepancies identified between patient and HPs perspectives have relevant implications for COS development and clinical practice. Virdum (2023) notes that the relative attention given to different domains by Patient-Reported Experience Measures (PREMs) and PROMs developers does not always reflect how patients and families weigh these domains in terms of importance; rather, it may be based on what is perceived as easier to collect or more clinically significant as defined by professionals or health organizations [38]. Our results suggest that it is crucial to adopt a more holistic approach in developing COS for perioperative care that balances technical medical outcomes with patient-centered quality of life measures. This aligns with the positive association between patient experiences and clinical effectiveness and safety described by Doyle et al. (as cited in Gobbo, 2020) [26]. This highlights the need for concrete actions, such as ensuring a more balanced representation of patients and healthcare professionals in expert panels involved in the development of Core Outcome Sets and Core Measurement Sets. Additionally, it may be necessary to make the inclusion of outcomes valued by patients a mandatory criterion in the prioritization process.

## Limitations and strengths of the study

This study had some limitations that should be considered when interpreting the results. Recruitment difficulties led to a smaller sample size than initially planned, potentially limiting the data saturation and reducing participant heterogeneity in terms of relevant expertise and professional background. The use of English as the primary language for discussions, since it was not the native language for most participants, may have affected the depth and nuance of expressions. Group dynamics presented challenges, with some participants systematically intervening more than others possibly due to cultural differences, personal temperament and the online. The presence of individuals from diverse countries and cultures, while enriching the study, also introduced potential variability in perspectives and experiences that may not be fully accounted for in the analysis. Additionally, the moderators’ limited experience in conducting focus groups could have influenced the flow and direction of discussions. These factors collectively may have impacted the comprehensiveness of the data collected and should be considered when interpreting the study’s findings.

This qualitative study demonstrates several notable strengths in exploring the discrepancies between HPs’ and patients’ perspectives on perioperative outcome measures. By employing focus groups, the research captures rich, in-depth insights from both patients and HPs, allowing for a thorough comparison of viewpoints. The use of Thematic Qualitative Text Analysis provided a robust framework for systematically identifying key themes and emergent codes, integrating both deductive and inductive approaches [16]. This method not only captured predefined categories but also revealed new insights that emerged directly from participants’ experiences. By incorporating both perspectives, the research offers a deeper understanding of communication gaps, care discrepancies, and varying priorities.

## Conclusion

This study highlights the critical importance of incorporating patient perspectives into the development of Core Outcome Sets for Patient Safety in Perioperative Care. The relevant differences in priorities and concerns between patients and HPs underscore the need for a more inclusive and patient-centered approach. Improving communication, addressing knowledge gaps, and considering emotional aspects along with quality of life factors are essential steps toward enhancing outcomes and patient satisfaction. Future COS developers should actively seek to integrate these diverse perspectives to ensure that prioritized outcomes are truly relevant to all stakeholders involved in perioperative care, thus fostering a more patient-focused healthcare system, ultimately improving the quality of care and patient experiences and safety.

## Supporting information

S1 File Appendices. (PDF)

S2 File Database used to build graphs. (XLSX)

## References

[1] de Oliveira HCSA, Marques RR, dos Santos Curado MA, Gaspar MFM, dos Santos Sousa PJ. Instruments for measuring incidents related to patient safety in the context of paediatric intensive care—protocol for a scoping review. Syst Rev. 2022;11(1):6–13. 35078533 10.1186/s13643-022-01888-6 PMC8790838

[2] O’Brien BC, Harris IB, Beckman TJ, Reed DA, Cook DA. Standards for reporting qualitative research: a synthesis of recommendations. Acad Med. 2014;89(9):1245–51. doi: 10.1097/ACM.0000000000000388 24979285

[3] Jha AK, Larizgoitia I, Audera-Lopez C, Prasopa-Plaizier N, Waters H, Bates DW. The global burden of unsafe medical care: analytic modelling of observational studies. BMJ Qual Saf. 2013;22(10):809–15. doi: 10.1136/bmjqs-2012-001748 24048616

[4] Commission of European Communities. Communication from the Commission to the European Parliament and the Council on Patient Safety, Including Prevention and Control of Healthcare-Associated Infections. 2008;9. Available from: http://ec.europa.eu/health/ph_systems/docs/patient_com2008_en.pdf

[5] de Vries EN, Ramrattan MA, Smorenburg SM, Gouma DJ, Boermeester MA. The incidence and nature of in-hospital adverse events: a systematic review. Qual Saf Health Care. 2008;17(3):216–23. doi: 10.1136/qshc.2007.023622 18519629 PMC2569153

[6] Schwendimann R, Blatter C, Dhaini S, Simon M, Ausserhofer D. The occurrence, types, consequences and preventability of in-hospital adverse events – a scoping review. 2018;1–13. 10.1186/s12913-018-3335-z PMC6032777 29973258

[7] Sousa P, Uva AS, Serranheira F, Uva MS, Nunes C. Patient and hospital characteristics that influence incidence of adverse events in acute public hospitals in Portugal: a retrospective cohort study. Int J Qual Health Care. 2018;30(2):132–7. doi: 10.1093/intqhc/mzx190 29309608 PMC5890867

[8] Affairs D of E and S. United Nations. Sustainable Development. Available from: https://sdgs.un.org/

[9] Forster AJ, Dervin G, Martin Jr. C, Papp S. Improving patient safety though th systematic evaluation of patient outcomes. 2012;55. 10.1503/cjs.007811 PMC3506692 23177520

[10] World Health Organization. Towards eliminating avoidable harm health care [Internet]. Global patient safety action plan 2021–2030. 2021. 1689–1699 p. Available from: https://www.who.int/teams/integrated-health-services/patient-safety/policy/global-patient-safety-action-plan

[11] Valli C, Schäfer WLA, Bañeres J, Groene O, Arnal-Velasco D, Leite A, et al. Improving quality and patient safety in surgical care through standardisation and harmonisation of perioperative care (SAFEST project): A research protocol for a mixed methods study. PLoS One. 2024;19(6):e0304159. doi: 10.1371/journal.pone.0304159 38870215 PMC11175406

[12] Williamson PR, Altman DG, Bagley H, Barnes KL, Blazeby JM, Brookes ST. The COMET Handbook: Version 1.0. Trials. 2017;18(Suppl 3):1–50. 28681707 10.1186/s13063-017-1978-4 PMC5499094

[13] Dinis-Teixeira JP, Nunes AB, Leite A, Schäfer WLA, Valli C, Martínez-Nicolas I, et al. Moving towards a core measures set for patient safety in perioperative care: An e-Delphi consensus study. PLoS One. 2024;19(10):e0311896. doi: 10.1371/journal.pone.0311896 39441853 PMC11498713

[14] Document P, Version R. Improving quality and patient SAFE ty in surgical care through ST andardisation and harmonisation of perioperative care in Europe D6. 1: Core Outcome Set for patient safety in perioperative care. 2023.

[15] Knapp A, Harst L, Hager S, Schmitt J, Scheibe M. Use of Patient-Reported Outcome Measures and Patient-Reported Experience Measures Within Evaluation Studies of Telemedicine Applications: Systematic Review. J Med Internet Res. 2021;23(11):e30042. doi: 10.2196/30042 34523604 PMC8663685

[16] Onwuegbuzie AJ, Dickinson WB, Leech NL, Zoran AG. A Qualitative Framework for Collecting and Analyzing Data in Focus Group Research. International Journal of Qualitative Methods. 2009;8(3):1–21. doi: 10.1177/160940690900800301

[17] Rabiee F. Focus-group interview and data analysis. Proc Nutr Soc. 2004;63(4):655–60. doi: 10.1079/pns2004399 15831139

[18] Tong A, Sainsbury P, Craig J. Consolidated criteria for reporting qualitative research (COREQ): a 32-item checklist for interviews and focus groups. Int J Qual Health Care. 2007;19(6):349–57. doi: 10.1093/intqhc/mzm042 17872937

[19] Nunes AB, Teixeira JP, Leite A, Schäfer W, Valli C, Martínez-Nicolas I, et al. Core measure set for patient safety in perioperative care: A clinical practice-oriented consensus study. Int J Public Health. 2026;71:1609159. doi: 10.3389/ijph.2026.1609159 PMC12989447 41847418

[20] Willemsen RF, Aardoom JJ, Chavannes NH, Versluis A. Online synchronous focus group interviews: Practical considerations. Qualitative Research. 2022;23(6):1810–20. doi: 10.1177/14687941221110161

[21] Sónia Maria Ferreira Dias AG. Introdução à investigação qualitativa em saúde pública [Internet]. Coimbra: Almedina; 2019. 178 p. Available from: http://id.bnportugal.gov.pt/bib/bibnacional/2006718

[22] Dong L. Qualitative text analysis: A guide to methods, practice & using software. Technical Communication. 2015;62.

[23] 2016/679. R (EU). General Data Protection Regulation. 2016; Available from: http://www.bloomsburycollections.com/book/fundamental-texts-on-european-private-law-1

[24] Tylee MJ, Rubenfeld GD, Wijeysundera D, Sklar MC, Hussain S, Adhikari NKJ. Anesthesiologist to Patient Communication: A Systematic Review. JAMA Netw Open. 2020;3(11):e2023503. doi: 10.1001/jamanetworkopen.2020.23503 33180130 PMC7662141

[25] Alnashri YM, Alfaqih OY, Buhaliyqh MA, Mossery RA, Alamri IR, Mahfouz NA, et al. Patient Satisfaction and Its Predictors With Perioperative Anesthesia Care at Two General Hospitals in Southwestern Saudi Arabia. Cureus. 2023;15(1):e33824. doi: 10.7759/cureus.33824 36819326 PMC9930371

[26] Gobbo M, Saldaña R, Rodríguez M, Jiménez J, García-Vega MI, de Pedro JM, et al. Patients’ Experience and Needs During Perioperative Care: A Focus Group Study. Patient Prefer Adherence. 2020;14:891–902. doi: 10.2147/PPA.S252670 32546983 PMC7266520

[27] Dy SM. Patient Safety and End-of-Life Care: Common Issues, Perspectives, and Strategies for Improving Care. Am J Hosp Palliat Care. 2016;33(8):791–6. doi: 10.1177/1049909115581847 25877945

[28] Gleason. 乳鼠心肌提取 HHS Public Access. Physiol Behav. 2019;176(5):139–48.

[29] Chegini Z, Arab-Zozani M, Janati A. Patient and Health Professional Perspectives about Engaging Patients in Addressing Patient Safety: A Systematic Review Protocol. Open Access Maced J Med Sci. 2019;7(9):1561–5. doi: 10.3889/oamjms.2019.280 31198473 PMC6542410

[30] Ebben RHA, Vloet LCM, Speijers RF, Tönjes NW, Loef J, Pelgrim T, et al. A patient-safety and professional perspective on non-conveyance in ambulance care: a systematic review. Scand J Trauma Resusc Emerg Med. 2017;25(1):71. doi: 10.1186/s13049-017-0409-6 28716132 PMC5513207

[31] Matthias AT, Samarasekera DN. Preoperative anxiety in surgical patients - experience of a single unit. Acta Anaesthesiol Taiwan. 2012;50(1):3–6. doi: 10.1016/j.aat.2012.02.004 22500906

[32] Avalere Health. Patient-Centered Value Frameworks Version 1.0: Methodology Report. Washington (DC): Avalere Health; 2017. Available from: https://advisory.avalerehealth.com/wp-content/uploads/2018/06/1494508191_201705010_PPVF_Version_1.0_Methodology_Report_Final.pdf

[33] Young B, Bagley H. Including patients in core outcome set development: issues to consider based on three workshops with around 100 international delegates. Res Involv Engagem. 2016;2:25. doi: 10.1186/s40900-016-0039-6 29507761 PMC5831887

[34] Barrington H, Young B, Williamson PR. Patient participation in Delphi surveys to develop core outcome sets: systematic review. BMJ Open. 2021;11(9):e051066. doi: 10.1136/bmjopen-2021-051066 34475183 PMC8413947

[35] Gorst SL, Young B, Williamson PR, Wilding JPH, Harman NL. Incorporating patients’ perspectives into the initial stages of core outcome set development: a rapid review of qualitative studies of type 2 diabetes. BMJ Open Diabetes Res Care. 2019;7(1):e000615. doi: 10.1136/bmjdrc-2018-000615 30899531 PMC6398822

[36] Kearney A, Williamson PR, Dodd S. A review of core outcome sets (COS) developed for different settings finds there is a subset of outcomes relevant for both research and routine care. J Clin Epidemiol. 2024;173:111440. doi: 10.1016/j.jclinepi.2024.111440 38936556

[37] Dean S, Al Sayah F, Johnson JA. Measuring value in healthcare from a patients’ perspective. J Patient Rep Outcomes. 2021;5(Suppl 2):88. doi: 10.1186/s41687-021-00364-4 34637001 PMC8511223

[38] Virdun C, Garcia M, Phillips JL, Luckett T. Description of patient reported experience measures (PREMs) for hospitalised patients with palliative care needs and their families, and how these map to noted areas of importance for quality care: A systematic review. Palliat Med. 2023;37(7):898–914. doi: 10.1177/02692163231169319 37092501 PMC10320712

