# Supplementary material for: Understanding discrepancies in perceived importance of patient safety measures between patients and healthcare professionals in perioperative care: An exploratory study
Source: PLoS One. 2026 Mar 17;21(3):e0344802. doi: 10.1371/journal.pone.0344802 (PMC12994851; doi:10.1371/journal.pone.0344802)
Supplement: S1 File — (PDF) [file pone.0344802.s001.pdf]

APPENDICES

Appendix 1- Categorized COS measures

Communication and Information Exchange in Patient Diagnostic	Long-Term Outcomes and Recovery and Functional Status	Psychological and Emotional Recovery	Pain Management and Well-being of patients and caregivers
○ Proportion of prospective surgical patients that are the target of a cardiovascular risks' discussion preoperatively<br>○ Proportion of prospective surgical patients that undergo echocardiography assessment preoperatively<br>○ Surgical team satisfaction	○ Time to recurrence or progression of disease<br>○ Long-term 'disease-specific' survival<br>○ Overall long-term survival<br>○ Cure of underlying disease.<br>○ Short-term and long-term level of functional dependence.<br>○ Functional status.<br>○ Time to resume to normal level of mobility.<br>○ Time to resume work and other usual life roles.<br>○ Postoperative sleep quality.<br>○ Physical recovery.<br>○ Time to gastro-intestinal recovery.<br>○ Time to mobilization.<br>○ Physiotherapy tolerance.<br>○ Overall recovery.<br>○ Postoperative quality of recovery.	○ Postoperative awareness.<br>○ Postoperative neuropsychologic performance deterioration.<br>○ Postoperative mental health symptoms.<br>○ Cognitive recovery.<br>○ Psychological recovery.	○ Postoperative endometritis<br>○ Wound healing time<br>○ Length of stay in Post-Anesthesia Recovery Area<br>○ Postoperative composite Pharyngolaryngeal adverse events<br>○ Postoperative shivering.<br>○ Post-discharge nausea and vomiting.<br>○ Postoperative nausea and vomit.<br>○ Postoperative nausea and vomit severity.<br>○ Need for rescue antiemetics.<br>○ Postoperative fatigue.<br>○ Opioid-related side-effects.<br>○ Time to first analgesic request.<br>○ Time to lowest pain score.<br>○ Analgesic consumption.<br>○ Independence from opioids.<br>○ Chronic pain.<br>○ Patient well-being.<br>○ Perioperative thermal comfort.<br>○ Caregiver satisfaction.<br>○ Long-term impact on relatives and/or caregivers.<br>○ Pain (at rest or on movement).<br>○ Pain-associated fear.

Appendix 2- Focus Group Discussion Guide

Focus Group Discussion Guide

Theme: Understanding Discrepancies in Perceived Importance of Patient Safety Indicators between Patients and Healthcare Professionals in Perioperative Care within the SAFEST Project - an exploratory study

Introduction

1.Introduction

"Good [morning/afternoon/evening], everyone. Thank you for being here today. My name is Maria and I'm joined by my colleague Ana. This focus group is being carried out for the SAFEST project short name for "**Improving quality and patient safety in surgical care through standardization and harmonization of perioperative care in Europe**", funded by the EU Horizon Research Program, focuses on a Core Outcome Set (COS) for Patient Safety in Perioperative Care, aiming to standardize practices and reduce perioperative complications.

I'll be leading our discussion, guiding our conversation with questions and ensuring everyone gets an opportunity to contribute. Ana will assist me in managing the discussion, note-taking, and handle logistics. To capture all valuable insights, we'll be recording this session. Our discussion will last approximately two hours."

Our goal today is to gather insights to understand the discrepancy between patient and healthcare professional perspectives on perioperative outcome indicators selected.

2. Consent form and demographic questionnaire – 10 minutes standby

"Before we begin the discussion, I would like you to review the consent form and the demographic questionnaire sent to you by email. Did everyone complete both things? I can give you 5 min if you didn't, I just sent it to the group chat.

Are there any questions about these?

The consent form will be our record that you agreed to participate in the focus group, you agree to the recording, and you understand that we will keep information confidential. We need a signed consent form from each participant.

The consent form also says that everyone in the room should respect confidentiality. If you know each other, we ask that you not talk about specific individuals and the information they shared. It is fine to talk about the discussion, but not to identify who said what.

The questionnaire will give us some information about your background.
We will combine the information from all participants and use it to describe the whole group. We will never use any identifying information about you in our reports.

3. Confidentiality

"All the information we collect here today is confidential. We will use the information you provide, but we will not identify any of you in anything we do related to this meeting. For example, we will not use your name, address, or any other identifying information in reports or other materials related to this focus group. The information collected will be safely kept and only members of the research team with permission will be able to access it. Partial de-identification data might be included in open-access scientific publications. When the project is finished, which is expected to happen in June 2026, the data that was gathered will be permanently deleted."

Focus group guidelines

" Allow me to recap some of the focus group's rules before we get started.
A few specific topics will be our focus. We are interested in what everyone has to say about them. Please speak up if you have a different opinion or if someone brings up an idea that you would like to elaborate on. Sometimes I may have to interrupt the discussion to bring us back to the topic or to move on to another question or topic, to make sure that we cover everything on our agenda.
We will follow several practical guidelines during this session:

- We want everyone to express your opinions about the discussion topics. We are interested in different points of view. There are no right or wrong answers. We are not here to resolve any issues you may bring up or to reach agreement, we just want to understand your views. Please speak up if you have a different opinion or if someone brings up an idea that you would like to elaborate on.
- Give us only your first name or a nickname. No one needs identifying information about you. If you know each other, we ask that you agree to keep information confidential – if you discuss the things people said here, do not identify the people who said them.
- Feel free to agree or disagree with what other people say, while respecting their views.
- Please wait to be recognized by the moderator before speaking. Sometimes I may have to interrupt the discussion to bring us back to the topic or to move on to another question or topic, to make sure that we cover everything on our agenda

- Sometimes we will go around the table to share views on a topic. You can always “pass” if you prefer not to comment on that topic.

Do you have any questions so far?"

4. Purpose of the Focus Group Session

Now let me do a brief introduction about the theme.

It's crucial to acknowledge the significant impact of adverse events on patient safety in the healthcare landscape. These events, leading to injuries and loss of disability-adjusted life years (DALYs), present a pressing global public health concern.

At the core of SAFEST we developed of a Core Outcome Set (COS) for Patient Safety in Perioperative Care, which all of you were a part of. As you know a Core Outcome Set it’s a group of indicators selected by Healthcare Professionals and Patients used as a fundamental tool to standardize outcome measures.

During this process we found some discrepancies between Healthcare Professionals and Patients opinions. Patients often emphasized indicators related to their experienced outcomes and quality of life, differing from healthcare professionals' viewpoints.

These are the indicators that patients considered as more important. I will now display it on the screen for you to consult during the session.

(Tabela)

Understanding these discrepancies constitutes the core of this focus group. We want to explore why this happened.

20 MINUTES TOTAL

Focus group discussion

"Participant introductions: Now, let’s go around the room and have each of you introduce yourselves; give your first name or a nickname, and tell us a little bit about your interaction with the perioperative setting.

Now, let’s begin our focus group discussion. I will do some general questions and then we go through the indicators to explore disparities.

**General – 10 MINUTES**

Communication and Information Exchange in Patient Diagnostic	Long-Term Outcomes and Recovery and Functional Status	Psychological and Emotional Recovery	Pain Management and Well-being of patients and caregivers
○ Proportion of prospective surgical patients that are the target of a cardiovascular risks' discussion preoperatively<br>○ Proportion of prospective surgical patients that undergo echocardiography assessment preoperatively<br>○ Surgical team satisfaction	○ Time to recurrence or progression of disease<br>○ Long-term 'disease-specific' survival<br>○ Overall long-term survival<br>○ Cure of underlying disease.<br>○ Short-term and long-term level of functional dependence.<br>○ Functional status.<br>○ Time to resume to normal level of mobility.<br>○ Time to resume work and other usual life roles.<br>○ Postoperative sleep quality.<br>○ Physical recovery.<br>○ Time to gastro-intestinal recovery.<br>○ Time to mobilization.<br>○ Physiotherapy tolerance.<br>○ Overall recovery.<br>○ Postoperative quality of recovery.	○ Postoperative awareness.<br>○ Postoperative neuropsychologic performance deterioration.<br>○ Postoperative mental health symptoms.<br>○ Cognitive recovery.<br>○ Psychological recovery.	○ Postoperative endometritis<br>○ Wound healing time<br>○ Length of stay in Post-Anaesthesia Recovery Area<br>○ Postoperative composite Pharyngolaryngeal adverse events<br>○ Postoperative shivering.<br>○ Post-discharge nausea and vomiting.<br>○ Postoperative nausea and vomit.<br>○ Postoperative nausea and vomit severity.<br>○ Need for rescue antiemetics.<br>○ Postoperative fatigue.<br>○ Opioid-related side-effects.<br>○ Time to first analgesic request.<br>○ Time to lowest pain score.<br>○ Analgesic consumption.<br>○ Independence from opioids.<br>○ Chronic pain.<br>○ Patient well-being.<br>○ Perioperative thermal comfort.<br>○ Caregiver satisfaction.<br>○ Long-term impact on relatives and/or caregivers.<br>○ Pain (at rest or on movement).<br>○ Pain-associated fear.

Looking over all the indicators, why do you think there are different opinions between Healthcare Professional and Patients?

Can you give me some examples of social-cultural factors that can influence the choice of indicators to include in a Core Outcome Set?

Communication and Information Exchange in Patient Diagnostic – 10 MINUTES

Communication and Information Exchange in Patient Diagnostic
○ Proportion of prospective surgical patients that are the target of a cardiovascular risks' discussion preoperatively<br>○ Proportion of prospective surgical patients that undergo echocardiography assessment preoperatively<br>○ Surgical team satisfaction

In your perspective, why do you think patients prioritize the discussions on cardiovascular risks and the use of echocardiography assessments more than Healthcare Professionals?

In your opinion, why do patients value the satisfaction of the surgical team?

Can you give me a clinical example of these/that/on that happened?

Long-Term Outcomes Recovery and Functional Status – 20 MINUTES

Long-Term Outcomes and Recovery and Functional Status
○ Time to recurrence or progression of disease<br>○ Long-term 'disease-specific' survival<br>○ Overall long-term survival<br>○ Cure of underlying disease.<br>○ Short-term and long-term level of functional dependence.<br>○ Functional status.<br>○ Time to resume to normal level of mobility.<br>○ Time to resume work and other usual life roles.<br>○ Postoperative sleep quality.<br>○ Physical recovery.<br>○ Time to gastro-intestinal recovery.<br>○ Time to mobilization.<br>○ Physiotherapy tolerance.<br>○ Overall recovery.<br>○ Postoperative quality of recovery.

How would you define that a surgical procedure was successful?

Regarding these indicators (as…) why do think there are disparities between the opinion of patients and health professionals?

Long-Term Outcomes and Recovery and Functional Status
○ Time to recurrence or progression of disease<br>○ Long-term 'disease-specific' survival<br>○ Overall long-term survival<br>○ Cure of underlying disease.<br>○ Short-term and long-term level of functional dependence.<br>○ Functional status.<br>○ Time to resume to normal level of mobility.<br>○ Time to resume work and other usual life roles.<br>○ Postoperative sleep quality.<br>○ Physical recovery.<br>○ Time to gastro-intestinal recovery.<br>○ Time to mobilization.<br>○ Physiotherapy tolerance.<br>○ Overall recovery.<br>○ Postoperative quality of recovery.

And considering these ones? Why do think there are different opinions?

Psychological and Emotional Recovery – 20 MINUTES

Psychological and Emotional Recovery
○ Postoperative awareness.<br>○ Postoperative neuropsychologic performance deterioration.<br>○ Postoperative mental health symptoms.<br>○ Cognitive recovery.<br>○ Psychological recovery.

Regarding postoperative awareness, why might there be different perspectives between patients and health professionals?

Psychological and Emotional Recovery
○ Postoperative awareness.<br>○ Postoperative neuropsychologic performance deterioration.<br>○ Postoperative mental health symptoms.<br>○ Cognitive recovery.<br>○ Psychological recovery.

When it comes to postoperative neuropsychologic performance deterioration and postoperative mental health symptoms? Why do you think there are disparities between patients and health care opinions?

Psychological and Emotional Recovery
○ Postoperative awareness.<br>○ Postoperative neuropsychologic performance deterioration.<br>○ Postoperative mental health symptoms.<br>○ Cognitive recovery.<br>○ Psychological recovery.

And looking at indicators like cognitive recovery and psychological recovery. Why are there different opinions between healthcare professionals and patients?

Pain Management and Well-being of patients and caregivers – 20 MINUTES

○ Pain Management and Well-being of patients and caregivers
○ Postoperative endometritis<br>○ Wound healing time<br>○ Length of stay in Post-Anaesthesia Recovery Area<br>○ Postoperative composite Pharyngolaryngeal adverse events<br>○ Postoperative shivering.<br>○ Post-discharge nausea and vomiting.<br>○ Postoperative nausea and vomit.<br>○ Postoperative nausea and vomit severity.<br>○ Need for rescue antiemetics.<br>○ Postoperative fatigue.<br>○ Opioid-related side-effects.<br>○ Time to first analgesic request.<br>○ Time to lowest pain score.<br>○ Analgesic consumption.<br>○ Independence from opioids.<br>○ Chronic pain.<br>○ Patient well-being.<br>○ Perioperative thermal comfort.<br>○ Caregiver satisfaction.<br>○ Long-term impact on relatives and/or caregivers.<br>○ Pain (at rest or on movement).<br>○ Pain-associated fear.

Which factors do you think we can improve in perioperative environment to manage patients' and caregivers' anxiety?

Why do you think that are different perceptions about the overall well-being of the patient between healthcare professional and patients?

The importance of studying disparities in perspectives – 10 MINUTES

Communication and Information Exchange in Patient Diagnostic	Long-Term Outcomes and Recovery and Functional Status	Psychological and Emotional Recovery	Pain Management and Well-being of patients and caregivers
○ Proportion of prospective surgical patients that are the target of a cardiovascular risks' discussion preoperatively<br>○ Proportion of prospective surgical patients that undergo echocardiography assessment preoperatively<br>○ Surgical team satisfaction	○ Time to recurrence or progression of disease<br>○ Long-term 'disease-specific' survival<br>○ Overall long-term survival<br>○ Cure of underlying disease.<br>○ Short-term and long-term level of functional dependence.<br>○ Functional status.<br>○ Time to resume to normal level of mobility.<br>○ Time to resume work and other usual life roles.<br>○ Postoperative sleep quality.<br>○ Physical recovery.<br>○ Time to gastro-intestinal recovery.<br>○ Time to mobilization.<br>○ Physiotherapy tolerance.<br>○ Overall recovery.<br>○ Postoperative quality of recovery.	○ Postoperative awareness.<br>Postoperative neuropsychologic performance deterioration.<br>Postoperative mental health symptoms.<br>Cognitive recovery.<br>Psychological recovery.	○ Postoperative endometritis<br>Wound healing time<br>Length of stay in Post-Anaesthesia Recovery Area<br>Postoperative composite Pharyngolaryngeal adverse events<br>Postoperative shivering.<br>Post-discharge nausea and vomiting.<br>Postoperative nausea and vomit.<br>Postoperative nausea and vomit severity.<br>Need for rescue antiemetics.<br>Postoperative fatigue.<br>Opioid-related side-effects.<br>Time to first analgesic request.<br>Time to lowest pain score.<br>Analgesic consumption.<br>Independence from opioids.<br>Chronic pain.<br>Patient well-being.<br>Perioperative thermal comfort.<br>Caregiver satisfaction.<br>Long-term impact on relatives and/or caregivers.<br>Pain (at rest or on movement).<br>Pain-associated fear.

Do you consider it an advantage to include patients in the development of the Core Outcome Set (COS)? What, in your opinion, are the advantages of such inclusion?

Why do you think the professional group with perspectives most identical to patients was the anesthesia area?

Closing Remarks

"Thank you very much for participating in this focus group. The information you have provided has been very helpful. It will be used to improve the overall quality and safety of perioperative care through a more inclusive and comprehensive approach and improve the quality of future COS development.

Are there any questions that I can answer before we end the session?
Thank you again for your help. We really, really appreciate your time and your knowledge."

Appendix 3- Sociodemographic Questionnaire

Demographic Questionnaire

* Obrigatória

1. Email for Follow-Up *

2. Age Group *

- 18-34
- 35-54
- 55-74
- 75+
- Prefer not to answer

3. Gender *

- Male
- Female
- Non-binary
- Prefer not to answer

4. Level of education *

- Postgrade education
- Tertiary (higher) education
- Secondary school
- Primary school
- None/ Incomplete primary school
- Prefer no to answer

5. Ocupation *

- Healthcare professional
- Patient/ Patiente Representative
- Governmental agency representative
- Methodologist/Researcher
- Policymaker
- Private Sector Representative
- Regulatory agencies representative
- Guideline developer
- Other
- Prefer not to answer

6. Profession *

- Medical Doctor
- Nurse
- Quality Expert
- Hospital manager
- Physical therapist
- Other
- Prefer not to answer

7. Area of expertise *

- ◯ Anaesthesiology
- ◯ Sugery
- ◯ Primary Care
- ◯ Public Health
- ◯ Haematology
- ◯ Rehabilitation
- ◯ Other
- ◯ Geriatric
- ◯ Prefer not to answer

8. Submitterd to surgery ≤ 5 years ago *

- ◯ Yes
- ◯ No
- ◯ No, but a direct family member was
- ◯ Prefer not to answer

Este conteúdo não foi criado nem é aprovado pela Microsoft. Os dados que submeter serão enviados para o proprietário do formulário.

Microsoft Forms

Appendix 4 – Consent Form

SAFEST

Participant Consent Form

Focus groups with patients and health professionals in the scope of the study - Understanding Discrepancies in Perceived Importance of Patient Safety Indicators Between Patients and Healthcare Professionals in Perioperative Care within the SAFEST Project - an exploratory study

This focal group can take around 2 hours. The research team does not anticipate that there are any risks associated with your participation, but you have the right to stop the focus group or withdraw from the research at any time, without any consequences.

Thank you for agreeing to participate in this focus group as a part of the above study.

By signing this form:

- I confirm that I have read and understood the information letter and have had the opportunity to ask questions about the study.
- I understand that it is my own choice to participate in the study and that I can withdraw at any time, without giving any reason.
- I understand that any information I provide during the study will be kept confidential and securely stored.
- I understand that the answers I give will not affect the care or treatments I receive.
- I consent to participate in a foucus group. I know that the focus group will be recorded for the purpose of data analysis.
- I consent to storing the data (audio recording and transcribed text) on a secure drive. Only scientific researchers will have access to this drive.
- I agree that my responses may be used for research purposes (such as reports, publications and/or presentations). My name and other personal data will not be mentioned in this.
- I agree that every participants of focus grup should respect confidentiality.
- I have been able to ask any questions, and I understand that I am free to contact the researcher with any questions I may have in the future.
- I do not expect to receive any benefit or payment of my participation.
- I agree to participate in the study as described in the information letter.

Name of the participant: ____

Participant Signature: ____
Date:______________________

Researcher Signature: ____
Date: ______________________

Funded by
the European Union

Funded by the European Union. Views and opinions expressed are however those of the author(s) only and do not necessarily reflect those of the European Union or European Commission [granting authority]. Neither the European Union nor the granting authority can be held responsible for them. This project is financed by the European Union's Horizon Europe research and innovation programme under grant agreement No. 101057825 (SAFEST, Improving quality and patient SAFEty in surgical care through STandardisation and harmonisation of perioperative care in Europe).
